# Daily sedentary time and its association with risk for colorectal cancer in adults: A dose–response meta-analysis of prospective cohort studies

**DOI:** 10.1097/MD.0000000000007049

**Published:** 2017-06-02

**Authors:** Peng Ma, Yonggang Yao, Weili Sun, Shaojun Dai, Chuanxin Zhou

**Affiliations:** Department of Gastroenterology, The Second Clinical Medical College, Yangtze University, Jingzhou, Hubei Province, P.R. China.

**Keywords:** colorectal cancer, meta-analysis, prospective cohort studies, sedentary behaviors

## Abstract

Sedentary behavior is emerging as an independent risk factor for health. However, previous studies have indicated that sedentary behaviors are associated with the colorectal cancer risk, but presented controversial results.

Studies in PubMed and EMBASE were searched update to February 2017 to identify and quantify the potential dose–response association between daily sedentary time and colorectal cancer.

Twenty-eight eligible studies involving a total of 47,84,339 participants with 46,071 incident cases were included in this meta-analysis. Our results showed statistically significant association between prolong television viewing time and colorectal cancer (odds ratio [OR] 1.17, 95% confidence interval [CI] 1.09–1.24, *P* < .001). Additionally, we obtained the best fit at an inflection point of 2 hours per day in piecewise regression analysis, the summary relative risk (RR) of colorectal cancer for an increase of 2 hours per day television viewing was 1.07 (95% CI 1.05–1.10, *P* < .001). Furthermore, prolong occupational sitting time was correlated with a significantly higher risk of colorectal cancer (OR 1.15, 95% CI 1.08–1.22, *P* < .001), increasing 2 hours per day of occupational sitting time per day was associated with a 4% incremental in the risk of colorectal cancer (RR 1.04, 95% CI 1.01–1.08). Additionally, prolong total sitting time was associated with a significantly higher risk of colorectal cancer (OR 1.06, 95% CI 1.03–1.09, *P* < .001). Increasing 2 hours of total sitting time per day was associated with a 2% incremental in the risk of colorectal cancer (RR 1.02, 95% CI 1.01–1.06). Subgroup meta-analyses in study design, study quality, number of participants, and number of cases showed consistent with the primary findings.

Prolonged television viewing, occupational sitting time, and total sitting time are associated with increased risks of colorectal cancer.

## 1 Introduction

Colorectal cancer is the third most frequent malignancies cancer.^[1]^ The etiology of colorectal cancer involves both genetic and environmental factors. Therefore, understanding the impact of environmental factors on colorectal cancer will help to prevent colorectal cancer. Previous studies investigating have showed that sedentary behavior is emerging as an independent risk factor for health.^[2]^

Sedentary behaviors, defined as in sitting or reclining posture behavior characterized by <1.5 MET (multiples of the basal metabolic rate),^[3]^ such as television (TV) viewing, computer use, lying down, reading, or car-driving that are associated with increased all-cause mortality.^[4]^ This behavior is not a synonym for lack of physical activity, but considered distinct from inactivity. Adults should have in at least 150 minutes of moderate-intensity aerobic physical activity throughout the week in World Health Organization recommendation to reduce the risk of cardiovascular disease, type 2 diabetes, and certain cancers.^[5]^ However, only a small percentage of adults meet this recommendation because of social and technological changes.^[6]^

Previous studies have examined the association between sedentary behaviors and risk of colorectal cancer. However, the relationship between prolonged daily sedentary time and risk of colorectal cancer remains controversial.^[7–34]^ Additionally, daily sedentary time associated with colorectal cancer risk has not been quantitatively assessed in a meta-analysis. Thus, to clarify and quantitative assessed daily sedentary time in relation to colorectal cancer, we performed this comprehensive meta-analysis, aiming to identify the contribution of daily sedentary time to colorectal cancer and quantitatively assessed daily sedentary time in relation to colorectal cancer.

## 2 Methods

Our meta-analysis was conducted according to the Meta-analysis Of Observational Studies in Epidemiology (MOOSE) checklist.^[35]^ There are no ethical issues involved in our study for our data were based on published studies.

### 2.1 Search strategy

We included eligible studies to investigate the relationship between daily sedentary time and colorectal cancer risk in general adult populations. To develop a flexible, nonlinear, *r* meta-regression model, we required that an eligible study should have categorized into 3 or more levels. If multiple publications were available for a study, we included the longest follow-up study.

PubMed and EMBASE were searched for studies that were published update to February 2017, with keywords including “colorectal cancer” AND “television” or “TV” or “sedentary” or “sitting” or “screen time” or “occupational time.” We refer to the relevant original essays and commentary articles to determine further relevant research. Eligible study was also included through the reference lists of relevant review articles.

### 2.2 Study selection

Two independent researchers investigate information the correlation between daily sedentary time and colorectal cancer risk: outcome was colorectal cancer; the relative risks (RRs) at least 3 quantitative categories of daily sedentary time and colorectal cancer risk with 95% confidence intervals (CIs). Moreover, we precluded nonhuman studies, reviews, meta-analyses, editorials, and published letters. To ensure the correct identification of qualified research, the 2 researchers read the reports independently, and the disagreements were resolved through consensus by all of the authors.

### 2.3 Data extraction

Standardized data collection tables were used to extract data. Each eligible article information was extracted by 2 independent researchers. We extracted the following information: first author; publication year; mean value of age; country; study name; sex; cases and participants; the categories of tooth loss; RR or odds ratio (OR). We collected the risk estimates with multivariable-adjusted. The disagreements were resolved through consensus by all of the authors.

### 2.4 Statistical analysis

We pooled RR estimates to measure the association between daily sedentary time and colorectal cancer risk; the hazard ratios (HRs) were considered equivalent to the RR.^[36]^ Results in different subgroups of daily sedentary time and colorectal cancer risk were treated as 2 separate reports.

Due to different cut-off points for categories in the included studies, we performed a RR with 95% CI by increased 2 hours of sedentary time per day using the method recommended by Greenland, Longnecker, and Orsini et al. Dose of sedentary time used the median sedentary time. If the median sedentary time category was not available, the midpoint of the upper and lower boundaries was considered the dose of each category. In addition, restricted cubic splines (RCS) were used to evaluate the nonlinear association between sedentary time and colorectal cancer risk, with 3 knots at the 10th, 50th, and 90th percentiles of the distribution. A flexible meta-regression based on RCS function was used to fit the potential nonlinear trend, and generalized least-square method was used to estimate the parameters. This procedure treats statin use (continuous data) as an independent variable and log of RR of diseases as a dependent variable, with both tails of the curve restricted to linear. A *P* value is calculated for linear or nonlinear by testing the null hypothesis that the coefficient of the second spline is equal to 0.^[37]^

STATA software 12.0 (STATA Corp, College Station, TX) was used to evaluate the relationships between sedentary behavior and colorectal cancer. *Q* test and *I*^2^ statistic were used to assess heterogeneity among studies. Random-effect model was chosen if *P*_Q_ < .10 or *I*^2^ > 50%, otherwise, fixed-effect model was applied. Begg and Egger tests were used to assess the publication bias of each study. *P* < .05 was considered significant for all tests.

## 3 Results

### 3.1 Literature search results

**Figure 1:**
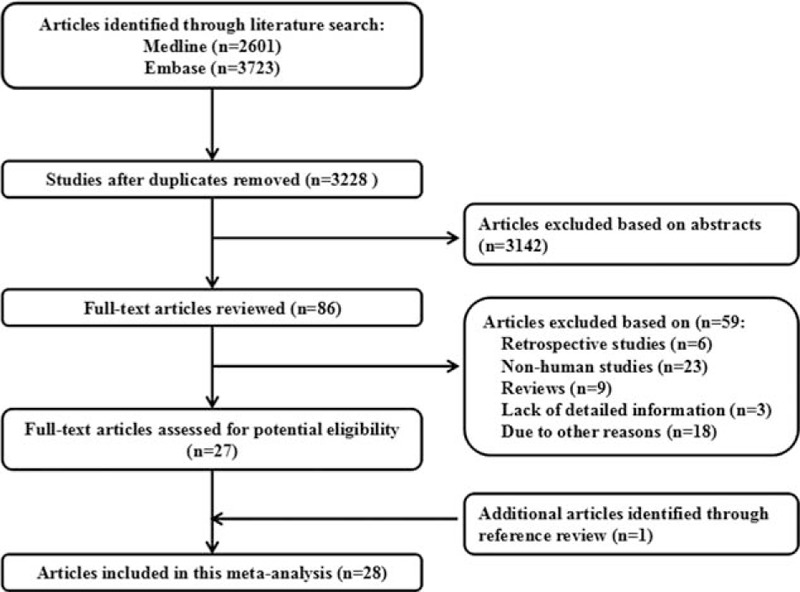
Flow diagram of the study selection process.

Figure 1 shows literature research and selection. A total of 2601 studies from PubMed and 3723 studies from EMBASE were selected. After exclusion of duplicates and studies that did not fulfill the inclusion criteria, 28 studies were chosen, and the data were extracted. These studies were published update to February 2017. Twelve reports for TV viewing time, 54 reports for occupational sitting time, and 6 reports for total sitting time.

### 3.2 Study characteristics

The characteristics of the included studies of sedentary behavior and colorectal cancer risk are shown in the Tables 1 and 2. Among the selected studies, 5 studies including 12 reports focused on TV viewing time and colorectal cancer, Twenty-three including 54 reports focused on TV occupational sitting and colorectal cancer, and 6 studies including 6 reports focused on total sitting time and colorectal cancer.

**Table 1:**
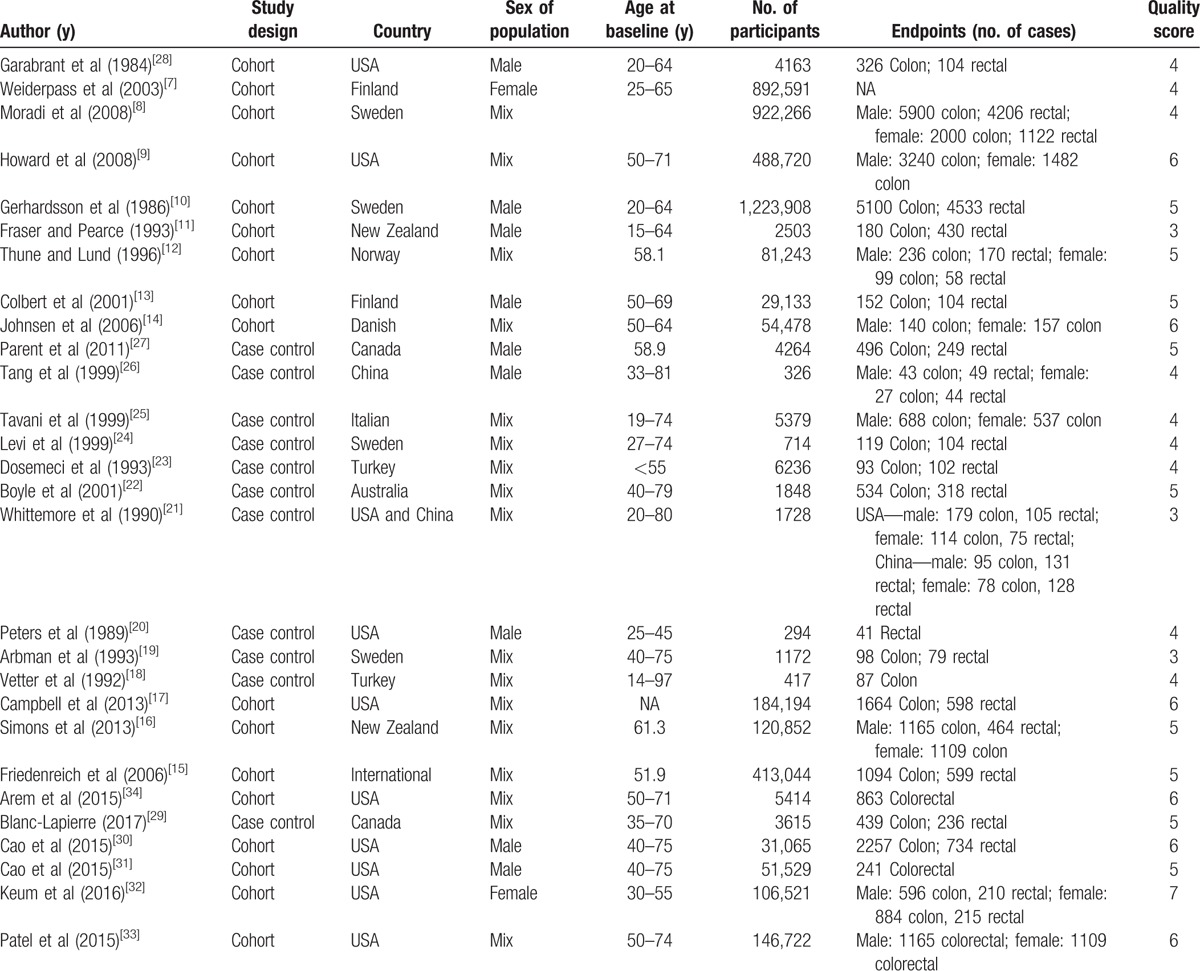
Characteristics of participants in included studies of daily sedentary time in relation to risk of colorectal cancer in adults.

**Table 2:**
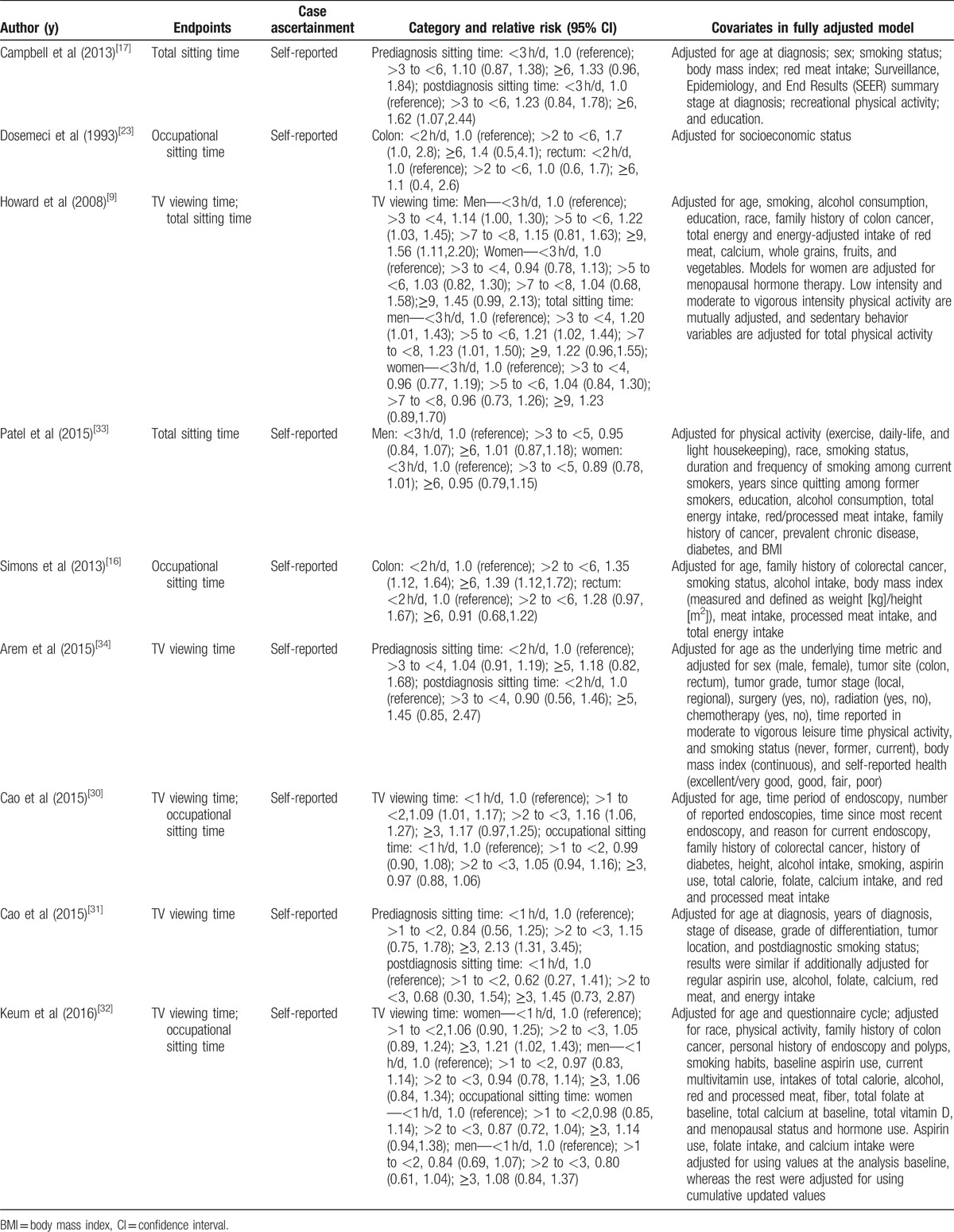
Outcomes and covariates of included studies of daily sedentary time in relation to risk of colorectal cancer in adults.

### 3.3 Daily TV viewing time and colorectal cancer

**Figure 2:**
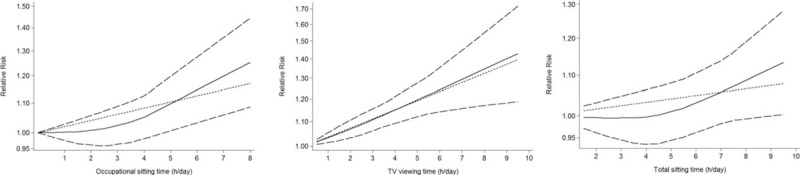
Dose–response relationship between daily sedentary time and risk of colorectal cancer in adults.

Twelve independent reports from 5 studies investigated the relationship between daily TV viewing time and the colorectal cancer risk. The results of TV viewing and colorectal cancer risk are shown in Table 3. Compared with the lowest daily TV viewing time, daily TV viewing time is significantly associated with a higher risk of colorectal cancer (pooled RR 1.17, 95% CI 1.09–1.24, *P* < .001). We found no evidence of between-study heterogeneity (*I*^2^ = 0.0%, *P* = .704) and we observed no evidence of publication bias (Egger asymmetry test, *P* = .185) (Table 4). Furthermore, prolonged daily TV viewing time was associated with a significantly higher risk in colon (RR 1.23, 95% CI 1.08–1.39) and rectal (RR 1.15, 95% CI 1.07–1.23) cancer. Additionally, we obtained the best fit at an inflection point of 2 hours per day in piecewise regression analysis, increase 2 hours of TV viewing per day was associated with a 7% increment in the risk of colorectal cancer (RR 1.07, 95% CI 1.05–1.10) (Fig. 2).

**Table 3:**
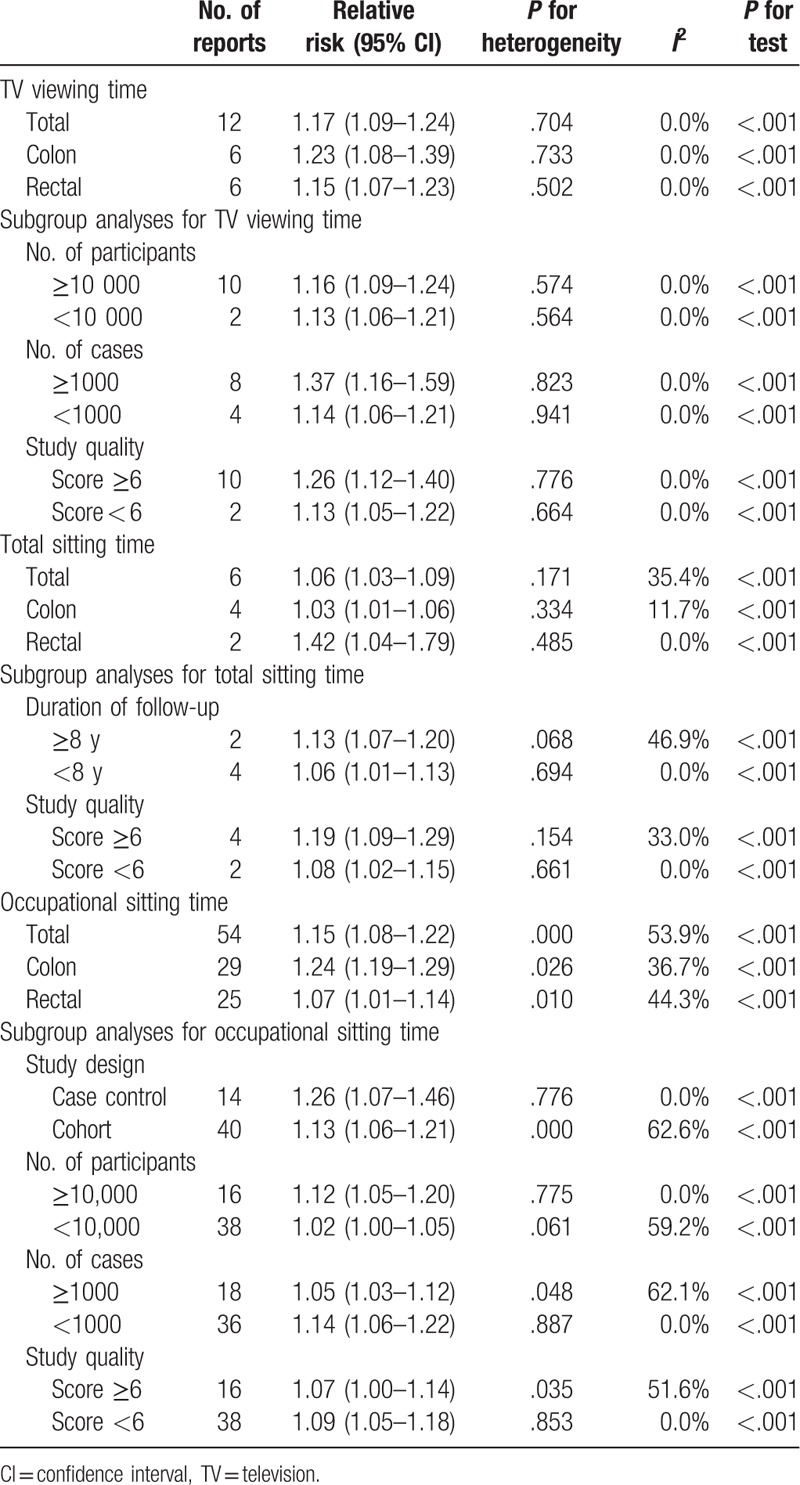
Stratified analyses of relative risk of colorectal cancer in adults.

**Table 4:**
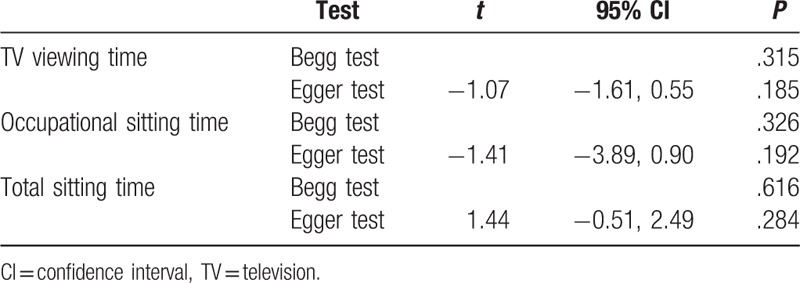
Publication bias analysis of the meta-analysis.

### 3.4 Daily total sitting time and colorectal cancer

Six independent reports from 3 studies investigated the relationship between daily total sitting time and the colorectal cancer risk. Compared with the lowest daily total sitting time, daily total sitting time is significantly associated with a higher risk of colorectal cancer (RR 1.06, 95% CI 1.03–1.09, *P* < .001)(Table 3). We found no evidence of between-study heterogeneity (*I*^2^ = 35.4%, *P* = .171) and we observed no evidence of publication bias (Egger asymmetry test, *P* = .284) (Table 4). Furthermore, prolonged daily total sitting time was associated with a significantly higher risk in colon (RR 1.03, 95% CI 1.01–1.06) and rectal (RR 1.42, 95% CI 1.04–1.79) cancer. In addition, increased 2 hours of total sitting time per day was associated with a 2% increment in the risk of colorectal cancer (RR 1.02, 95% CI 1.01–1.06) (Fig. 2).

### 3.5 Daily occupational sitting time and colorectal cancer

Fifty-four independent reports from 21 studies investigated the association between daily total sitting time and the colorectal cancer risk. Compared with the lowest daily occupational sitting time, daily occupational sitting time is significantly associated with a higher risk of colorectal cancer 1.15 (95% CI 1.08–1.22, *P* < .001) (Table 3). We found evidence of between-study heterogeneity (*I*^2^ = 53.9%, *P* = .000), but we observed no evidence of publication bias (Egger asymmetry test, *P* = .192) (Table 4). Furthermore, prolonged daily occupational sitting time was associated with a significantly higher risk in colon (RR 1.24, 95% CI 1.19–1.29) and rectal (RR 1.07, 95% CI 1.04–1.79) cancer. In addition, increased 2 hours of total sitting time per day was associated with a 3% increment in the risk of colorectal cancer (RR 1.03, 95% CI 1.01–1.14) (Fig. 2).

### 3.6 Subgroup analyses

Subgroup meta-analyses in study design, study quality, number of participants, and number of cases showed consistency with the primary findings (Table 3).

### 3.7 Publication bias

Each study in this meta-analysis was performed to evaluate the publication bias by both Begg funnel plot and Egger test. *P* > .05 was considered no publication bias. The results show no obvious evidence of publication bias was found in the associations between TV viewing, total sitting time, and occupational sitting time and risk for colorectal cancer (Table 4).

## 4 Discussion

Prolonged sedentary time association with colorectal cancer is biologically plausible. Sedentary behavior is often accompanied by concurrent intake of foods and food advertising on TV may promote an unhealthy diet.^[38–40]^ Keum et al^[32]^ and Johnsen et al^[14]^ found that sedentary time did not associate with colorectal cancer risk. However, other articles hold the opposite view on TV viewing time and colorectal cancer risk. Collectively, these data suggest that sedentary time play an important role in colorectal cancer, but it may act diversely. Thus, we perform this meta-analysis to investigate the pooled effect size of this association.

To our knowledge, this is the first study to identify and quantify the potential dose–response association between daily sedentary time and colorectal cancer in adults in a large cohort. The primary finding in our meta-analysis is that prolong TV viewing time, total sitting time, and occupational sitting time are associated with increased colorectal cancer risk. Increasing 2 hours per day of TV viewing is associated with a 7% incremental risk of colorectal cancer, increasing 2 hours per day of occupational sitting time is associated with a 4% incremental risk of colorectal cancer, and increasing 2 hours per day of total sitting time is associated with a 2% incremental risk of colorectal cancer. Furthermore, a publication bias existed in recessive model, indicating number of studies in the current meta-analysis. Taken together, our meta-analysis stably showed significant association between prolong TV viewing time, total sitting time, and occupational sitting time and colorectal cancer risk

We performed this comprehensive meta-analysis; however, some limitations must be considered in the current meta-analysis. First, different sex of population should be included in this meta-analysis to explore the impact of different sex of population on sedentary time and colorectal cancer in adults. Second, we only selected literature that was written in English, which may have resulted in a language or cultural bias; other languages should also be chosen in the future. Third, there might be insufficient statistical power to check the association.

In conclusion, our meta-analysis suggests prolonged TV viewing, occupational sitting time, and total sitting time was independently associated with deleterious colorectal cancer in adults. However, large sample size, different ethnic population, and different sex of population are warranted to validate this association.
